# CoMentG: comprehensive retrieval of generic relationships between biomedical concepts from the scientific literature

**DOI:** 10.1093/database/baae025

**Published:** 2024-04-02

**Authors:** Jorge Novoa, Javier López-Ibáñez, Mónica Chagoyen, Juan A G Ranea, Florencio Pazos

**Affiliations:** Computational Systems Biology, National Center for Biotechnology (CNB-CSIC), c/ Darwin, 3., Madrid 28049 , Spain; Computational Systems Biology, National Center for Biotechnology (CNB-CSIC), c/ Darwin, 3., Madrid 28049 , Spain; Computational Systems Biology, National Center for Biotechnology (CNB-CSIC), c/ Darwin, 3., Madrid 28049 , Spain; Department of Molecular Biology and Biochemistry, University of Málaga, Avda. Cervantes, 2., Málaga 29071, Spain; CIBER de Enfermedades Raras (CIBERER), Instituto de Salud Carlos III, Madrid, Spain; Institute of Biomedical Research in Malaga and platform of nanomedicine (IBIMA platform BIONAND), Malaga 29071, Spain; Spanish National Bioinformatics Institute (INB/ELIXIR-ES), Barcelona 08034, Spain; Computational Systems Biology, National Center for Biotechnology (CNB-CSIC), c/ Darwin, 3., Madrid 28049 , Spain

## Abstract

The CoMentG resource contains millions of relationships between terms of biomedical interest obtained from the scientific literature. At the core of the system is a methodology for detecting significant co-mentions of concepts in the entire PubMed corpus. That method was applied to nine sets of terms covering the most important classes of biomedical concepts: diseases, symptoms/clinical signs, molecular functions, biological processes, cellular compartments, anatomic parts, cell types, bacteria and chemical compounds. We obtained more than 7 million relationships between more than 74 000 terms, and many types of relationships were not available in any other resource. As the terms were obtained from widely used resources and ontologies, the relationships are given using the standard identifiers provided by them and hence can be linked to other data. A web interface allows users to browse these associations, searching for relationships for a set of terms of interests provided as input, such as between a disease and their associated symptoms, underlying molecular processes or affected tissues. The results are presented in an interactive interface where the user can explore the reported relationships in different ways and follow links to other resources.

**Database URL**: https://csbg.cnb.csic.es/CoMentG/

## Introduction

Modern biomedicine is characterized by the accumulation of massive amounts of data whose mining could provide valuable knowledge with eventual practical applications for disease diagnosis and treatment. That mining is hindered by the still scarce representation of these data using formal vocabularies and ontologies, which is necessary for relating conceptual entities between different resources and, in general, for representing the information in a computer-tractable way. Elementary processes such as retrieving a comprehensive list of associations between complex diseases and their reported symptoms or affected biological processes, given in terms of formal identifiers of controlled vocabularies, are not trivial, and in many cases, these have to be manually generated or inferred/predicted from indirect evidences.

There are many initiatives aimed at developing and maintaining these standard vocabularies for representing diverse biological concepts. One of the more accepted and widely used is that generated by the Gene Ontology (GO) consortium for representing different biological functions (1). GO defines a set of standard terms, associated with the corresponding identifiers, for representing different functional aspects of gene products, such as their molecular functions, the biological processes they are involved in and the cellular compartments (CCs) where they perform these functions. Having the functional information for two genes given in terms of GO identifiers allows, for example, quantifying their ‘functional similarity’, which could not be done with just textual descriptions of the genes’ functions. The ‘*de facto*’ standard vocabulary to describe the clinical manifestations and phenotypical abnormalities associated with diseases is that developed by the Human Phenotype Ontology (HPO) (2). Human diseases are represented in many controlled vocabularies, including the MONDO Disease Ontology (3) and the Disease Ontology (DOID) (4). Tissues, body parts and cell types can also be represented by controlled vocabularies, such as Uber-anatomy ontology (UBERON) (5) and Cell Ontology (CL) (6).

Defining relationships between the terms in these and other standard vocabularies would make it possible to build a large network of relationships that would comprise a lot of biomedicine-related information. While some of these linkages are available for some pairs of biomedical concepts, many are not available (7), and many others are based on indirect evidences or predictions [e.g. linking GO functions and HPO symptoms or diseases based on shared annotated genes (8)]. Indeed, it is for the relationships between pathology-related concepts and molecular functions/biological processes where the situation is worse, despite the importance of these connections for linking, for example, diseases with their underlying molecular mechanisms.

While manually curated annotation is the best alternative to generate these linkages, it has obvious limitations for being applied at a large scale. As many of those relationships are described in the scientific literature in textual form, without providing identifiers of these controlled vocabularies, one alternative is to use text-mining approaches to extract them and present them in terms of these formal IDs. We have developed such an approach for extracting from the scientific literature relationships between generic biomedical terms (7). The method looks for significant co-mentions in the PubMed abstract corpus between the textual descriptions of two terms (including their synonyms). In a proof of concept, we had previously applied it for extracting relationships between three types of biomedical entities (diseases, GO terms and HPO terms) generating, among other things, the first comprehensive set of relationships between GO terms and diseases/symptoms. We also compared some of these linkages with those available in manually curated resources and given in textual form, showing that, in general, our approach is able to capture a larger amount of meaningful relationships (7).

In this work, we apply that approach at a large scale in an attempt to cover all other aspects related to human pathologies. We run the method for a large set of ∼74 000 terms of nine controlled vocabularies covering the main aspects of human pathology: diseases (DOID and MONDO ontologies), biological processes, molecular functions and cell compartments (GO), clinical signs (HPO), cell types (CL), tissues and body parts (UBERON), bacteria (MeSH terms (9) related to microorganisms) and chemical compounds [human-related metabolites from the Human Metabolome Database (HMDB) (10) and MeSH terms related to chemical compounds]. We obtained more than 7 million significant relationships (*P*-values ≤ 0.001) between these terms, comprising 17 different types of linkages between the 9 types of entities. Some of the types of linkages we generate here are totally new and not available in any other resource. Most of the others are not available using the same identifiers/vocabularies. For those available in other resources (using the same or different vocabularies), we retrieve more relationships in general. An interactive interface was developed where the user can look for relationships for a set of terms of interest, sort them by different parameters, follow up links to the terms’ specific databases to retrieve more information, perform automatic searches in Google and PubMed to dig into the meaning of the relationships and inspect the PubMed abstracts co-mentioning the terms, among other things, as well as exporting the table of relationships to perform further studies. The system includes an example-filled input form as well as a guided tutorial.

## Materials and methods

### Sets of biomedical terms

For the HPO, we took all the terms that have ‘phenotypic abnormality’ (HP:0000118) as an ancestor in the HPO hierarchy, in order to avoid terms not related to phenotypes or clinical signs, ending up in a final list of 16 218 terms From the DOID disease ontology, we retrieved 10 949 terms representing different diseases. From MONDO, we tried to exclude terms representing symptoms as these are already covered by HPO (see earlier). For that, we excluded the MONDO terms with equivalent HPO terms annotated, ending up in a final list of 21 926 terms. From GO, we retrieved the terms from the three sub-ontologies (‘molecular function’, ‘biological process’ and ‘CC’) associated with at least one human gene in the Gene Ontology Annotations resource (11), in an attempt to restrict to GO terms relevant to human, obtaining a final list of 18 892 terms. For the CL ontology, we took all CL:* terms, which are used for representing different human cell types (2532 terms). For the UBERON ontology, we took all UBERON:* terms, representing different tissues and body parts (14 273 terms). From the ‘C’ and ‘D’ subsets of the MeSH vocabulary, we retrieved the terms under the B03 category (‘bacteria’) of the hierarchy, as well as those associated with the semantic type T007 (‘bacterium’), in an attempt to get those terms of the MeSH generic vocabulary representing microorganisms. We imposed the additional constraint that the terms must be linked to National Center for Biotechnology Information (NCBI) Taxonomy IDs (12), ending up in a final list of 19 194 terms. From MeSH, we also retrieved the terms representing chemical compounds, as those under different ‘semantic types’ indicative of that, such as T109, T116 and T121, among others (2915 terms). Finally, from HMDB, we retrieve all entries (205 011), representing chemical compounds associated with human in different ways (metabolites, drugs, toxic compounds, etc.). Nevertheless, contrary to the other datasets, most of these chemical compounds are never mentioned in PubMed according to the searches we perform (see later), and hence our final list (HMDB compounds mentioned in PubMed) contains 24 880 terms. For all these datasets, we retrieved the terms’ names and synonyms annotated in the corresponding fields of the resources. For HMDB terms (chemical compounds), we excluded from their list of synonyms those associated with more than one compound. This is because some generic names are annotated as ‘synonyms’ in that resource, so that including them in the searches would retrieve not only the articles mentioning the specific term but also all those referring to the generic term.

### Detecting literature co-mentions

The process for detecting significant co-mentions in the scientific literature is described in detail in (7). In short, for each term, we search PubMed for its textual description (including synonyms combined with ‘OR’) using NCBI’s Entrez application programming interface (13), obtaining in this way the list of abstracts [given in terms of PubMed identifiers (PMIDs)] mentioning that term. As performing searches for two terms together (combined with ‘AND’) would be unfeasible for all pairs, we take the intersection between each term’s list of PMIDs as the set of articles mentioning the two together. For a given pair of terms, from the frequencies of articles mentioning each of them and those mentioning the two together, also taking into account the whole size of PubMed (∼36 million), we apply a hypergeometric test to obtain the *P*-value of the null hypothesis that the co-mention of both terms occurs by chance, as well as other figures indicative of the strength of the co-mention (7).

For two terms mentioned individually in *n*_1_ and *n*_2_ abstracts, respectively, and co-mentioned together in *b* abstracts within the whole PubMed corpus (*P* abstracts), that *P*-value would be calculated as


$$pval = 1 - \mathop \sum \limits_{i = 0}^{b} \frac{{\left( {\begin{array}{*{20}{c}}
{{n_1}}\\
i
\end{array}} \right)\left( {\begin{array}{*{20}{c}}
{P - {n_1}}\\
{{n_2} - i}
\end{array}} \right)}}{{\left( {\begin{array}{*{20}{c}}
P\\
{{n_2}}
\end{array}} \right)}}.$$

We also calculate a score of ‘string similarity’ between the textual descriptions of both terms (including their synonyms) (7) as terms with identical or very similar descriptions (such as the same concept represented in different ontologies) lead to trivial co-mentions that should be eventually discarded.

All the data retrieval and the bibliographic searches were performed between April and October 2022.

### Web interface

A web interface was developed where the user can search for one or more terms of interest in any of the nine categories and retrieve the associated terms in the others. This webserver was developed with HTML and CSS and uses JavaScript and PHP for the active parts. For the sorting table functionality, a modified version of the open-source sorttable.js package was used (https://www.kryogenix.org/code/browser/sorttable/). The interface was tested in all major web browsers and operative systems.

## Results

**Figure 1.**
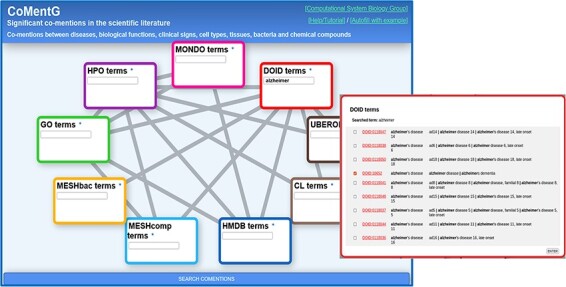
CoMentG main interface with the input form. In this example, the user is looking for relationships between ‘Alzheimer’s disease’ (DOID: 10652) and clinical signs (HPO), gene/protein functions (GO), cell types (CL) and chemical compounds (HMDB).

The procedure described in the Materials and Methods section led to a final set of 7 060 992 co-mention relationships (*P*-values ≤ 0.001) between terms of the nine resources. Not all possible pairs of resources were tested, but only 17 regarded as interesting (lines in Figure 1). For example, co-mentions between cell types (CL) and tissues (UBERON) or between HMDB and MeSH chemical compounds were not evaluated.

Supplementary File S1 contains a detailed list of 17 types of linkages, indicating the number of relationships retrieved for each type, as well as the number of terms involved. This table also shows information in other resources containing similar types of linkages, including information on whether these have been obtained from direct or indirect evidences (e.g. via shared genes) and whether they are given in terms of the same identifiers/vocabularies we use.

### Web interface

Figure 1 shows screenshots of the CoMentG web interface to the database and main input form. The text boxes for searching terms in the nine ontologies are highlighted with different colors. The lines connecting them indicate the 17 pairs of ontologies for which relationships between terms are available. The easiest way to use the interface is to search for one or more terms of interest in the boxes, select the line(s) corresponding to the relationship(s) we are interested in for these terms and press ‘SEARCH COMENTIONS’. The search boxes have an auto-completion feature, and more than one term matching the search criteria can be selected in order to retrieve relationships for all of them. This is useful, for example, when there is more than one term for the concept we are interested in (e.g. variants of a disease).

**Figure 2.**
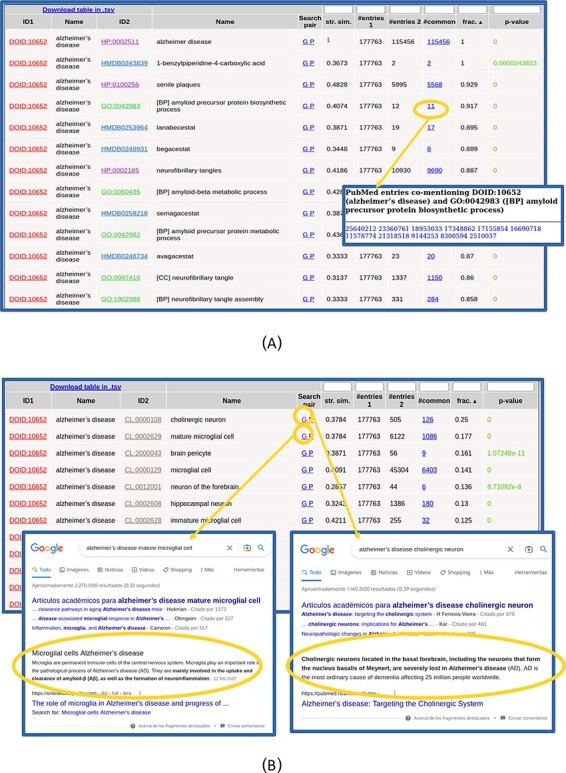
Table with the relationships found for ‘Alzheimer’s disease’. (A) Top of the table. The list of 11 PubMed entries co-mentioning Alzheimer’s disease and the biological process ‘amyloid precursor protein biosynthesis’ (GO: 0042983) is shown. (B) Table with the relationships with cell types (CL) only. Screenshots of Google result pages generated with the links for the top two relationships (‘Alzheimer’s disease’ with ‘cholinergic neurons’ and ‘mature microglial cells’) are shown.

The list of found relationships is shown in an interactive table (Figure 2). For each item in the list (pair of terms), the IDs and names of both terms are shown, being the first links to the entries for these terms in the corresponding resources. The ‘Search pair’ column contains links for performing text searches of both names together (without synonyms) in Google (‘G’) and PubMed (‘P’) in order to further investigate the relationship. The ‘string similarity’ between both names (including their synonyms, not shown in that table) is also shown, and cases of high similarity (≥0.7) are highlighted in red color. The next columns show the number of PubMed entries mentioning the first term, those mentioning the second, those mentioning both together and the ratio of these with respect to the minimum of the first two. The number of papers mentioning both terms is a link to the list of PMIDs where that co-mention was found (Figure 2A), which can be followed to the corresponding abstracts. The last column shows the *P*-value of the hypergeometric test for assessing the significance of the co-mention (see the Materials and Methods section). Only pairs with *P*-values ≤ 0.001 are shown, and those with *P*-values ≤ 1 × 10^−5^ are highlighted in green color. The table can be sorted by any column by clicking the corresponding header, and by default, it is sorted by the ratio of papers mentioning both terms with respect to the minimum of the papers mentioning each term individually. By sorting the table, it is possible, for example, to focus on the relationships with high values of a given parameter, those with best *P*-values or those involving terms of a given ontology (when more than one set of relationships were selected in the input form). The numerical columns have a text box in the header that allows filtering the list of relationships to those with certain values of that parameter: less than or equal to the entered value for *P*-value and string similarity and more or equal for the others. When a filter is applied, it is indicated in the column header. As the whole set of relationships matching the search criteria and filter is shown in a single table (i.e. without pagination), the browser’s ‘Search in page’ functionality can be used to look for particular terms in long lists.

Finally, there is a link to download the whole table in “tab separated values” (TSV) format in order to import it into an external spreadsheet program to further process it.

The web server includes a guided tutorial and an ‘Autofill with example’ option to test it right away.

### Example

This example shows how to use the server for retrieving relationships between Alzheimer’s disease and clinical signs, biological processes/molecular functions, cell types and chemical compounds, in order to have a picture of the different molecular aspects of this disease.

The first step is to look for the term corresponding to ‘Alzheimer’s disease’ on the ‘disease ontology’ (DOID) text box. For that, we start typing the term in the corresponding text box. If the term of interest does not show up in the auto-completion suggestions, pressing Enter shows a detailed list of matching terms (in this case variants of the disease) (Figure 1). This list includes the terms’ IDs (hyperlinked to the corresponding entries in the original resource to eventually retrieve more info), as well as their names and synonyms. In this list, it is possible to select one or more terms (in case, we want, for example, to retrieve relationships for more than one Alzheimer variant). In our case, we select only the generic entry for the disease (DOID: 10652), as we are not interested in any particular variant.

Once the term or terms of interest are in the corresponding box, we select the lines connecting that box with all the other datasets for which we want to retrieve relationships: in this case we select the lines connecting DOID (diseases) with HPO (symptoms, clinical signs), GO (gene functions), HMDB (human-related chemical compounds) and CL (cell types). Once the term(s) and the desired types of relationships are chosen, we press the [SEARCH COMENTIONS] button.

The co-mention relationships between the term(s) of interest and those terms in the other selected resources are shown in a table. The top of this list for our example is shown in Figure 2A. In this example, all the terms that show up in the list make sense considering what we intuitively know about this complex pathology and, together, they provide a comprehensive picture of its internal causes (molecular processes), external symptoms, cell types it affects and related drugs and chemical compounds. Regarding clinical signs (HPO), we see the typical molecular/physiological manifestations related to senile plaques, neurofibrillary tangles, cortical atrophy, hirano bodies, etc. as well as higher-level mental and behavioral symptoms (apathy, delusions, dementia, memory problems, etc.). Within the biological processes terms (GO[BP]), we find those related to β-amyloid metabolism and neurofibrillary tangles, among others. Regarding molecular functions (GO[MF]), we can see those related to binding to amyloid-beta and tau protein, among others. Also, the ‘CCs’ category of GO (GO[CC]) reflects what is known on this disease at the subcellular level, with terms such as gamma-secretase complex, alpha-ketoglutarate dehydrogenase complex and lewy bodies. For the chemical compounds (HMDB), the found relationships are mainly with drugs used for treating this disease, such as lanabecestat, begacestat or semagacestat, among others (Figure 2A). If one wants to inspect in detail the relationships with terms of a given ontology, it is better to select that alone in the input form, as done for the CL in Figure 2B. Regarding the cell types (CL terms), we see the typical brain-related cell types associated with this disease, with the first ones being ‘cholinergic neuron’ (CL:0000108), known to be severely affected in this disease, and ‘mature microglial cell’ (CL:0002629), which are involved in the uptake and clearance of β-amyloid. We can use the ‘G’ links to perform Google searches for these cell type relationships in order to dig into their involvement in Alzheimer (Figure 2B).

Note that the top scoring relationship found for DOID: 10652 (‘Alzheimer’s disease’) is a trivial one with HP:0002511 (‘Alzheimer’s disease’), as this term is also included in the HPO vocabulary (Figure 2A). These trivial cases can be detected by the ‘string similarity’ parameter (1.0 in this case, highlighted in red in Figure 2A) and eventually discarded introducing a threshold in the text box of the corresponding header.

## Discussion

Detecting co-mentions in the scientific literature is a widely used method for the automatic extraction of information and, eventually, its representation in a structured way (see for example (14–17)). Some of these systems include information on the type of linkage, and others are restricted to PubMed’s vocabulary and ontology (MeSH). While having its own drawbacks, described in detail later, the main advantage of our approach is that it is not tied to any particular ontology/vocabulary and that it requires a recurrent co-mention of two terms across a statistically significant number of documents in order to report a relationship.

The resource described here does not contain new or unknown relationships. Its utility resides in its coverage [i.e. it was generated scanning the whole scientific literature for (reported) relationships] and, more importantly, the fact that it delivers them using controlled vocabularies and standardized identifiers. This is fundamental for carrying out large-scale studies, for matching information between different resources and, in general, for representing the current biomedical knowledge in a computer-tractable way. The traditional way for describing relationships between biomedical concepts using standardized vocabularies and IDs is by manual annotation. The system presented here can help manual annotators supplying them with an initial set of relationships to check. Nevertheless, in the examples we have seen, the quality of the results is enough for using these relationships as they are. The system as it is can also be applied for detecting ‘self-relationships’, for example, a co-mention between two diseases indicative of comorbidity.

Although some of the types of relationships generated here are available (in a structured way) in other resources (leaving apart differences in coverage and methodology), many others are novel (Supplementary File S1). Among the novel relationships reported here, of special interest are the direct linkages of diseases with molecular functions (GO), cell types (CL) and tissues (UBERON) that, to the best of our knowledge, are not available anywhere despite their importance to perform systematic studies on the pathologies’ underlying molecular events and affected cells. There are resources linking these terms via indirect evidences, such as shared genes or chemical compounds. For example, Davis *et al*. (18) inferred GO—disease connections through the integration of GO gene annotations with the gene disease set from the Comparative Toxicogenomic Database (19). Similarly, HPO2GO (8) is based on co-annotations of the HPO and GO terms on the same genes/proteins. In comparison with HPO–GO pairs inferred from shared genes, our pairs point to more specific symptoms and biological functions. Our disease–GO pairs include many more diseases as our approach is not restricted to diseases with known associated genes. Moreover, these resources linking GO terms with other ontologies via shared genes do not include the CC sub-ontology due to the lack of direct gene annotations for it. For the other resources which contain similar linkages (using either indirect evidences or different vocabularies), in most cases, we retrieve more relationships involving more terms (Supplementary File S1).

This lack of similar resources makes it impossible to evaluate exhaustively all the linkages. Previously, we had evaluated some particular relationships for which equivalences exist in other resources, showing that, in general, our approach is able to capture a larger amount of meaningful relationships (7).

As it is now, the system reports generic relations between concepts but tells nothing about the meaning or cause for that relationship. For example, it cannot distinguish ‘positive’ from ‘negative’ relationships: e.g. Huntington’s disease is linked to ‘obesity’ as these two concepts are co-mentioned together but to state that Huntington’s disease ‘reduces’ obesity. Similarly, for the relationships between drugs and symptoms/diseases, we cannot disentangle those representing a drug’s intended effect from those representing side/adverse effects. The resource contains generic relationships without information on their meaning, and it is the user who has to interpret them in the context of the particular ontologies queried. For low-scale studies, the type of relationship can be easily grasped following the links to Google and PubMed in the web interface. We are already working on strategies to differentiate the type of relationship or retrieve more information on it.

The fact that we totally rely on the sets of synonyms provided by the different resources can also cause problems: for example, an artifactual relationship between ‘skin cancer’ (disease) and ‘dendritic spine neck’ (subcellular part) is reported due to the very generic term ‘neck’ being annotated in GO as a synonym for the second. What is described in the literature is the relationship between ‘skin cancer’ (disease) and ‘neck’ (body part), which is also indeed retrieved by the system. We are devising strategies for filtering out these artifacts due to ‘generic’ synonyms.

In general, this system contributes to the automatic retrieval of structured biological information from scientific text [‘biocuration’ (20, 21)]. For sure, the recent advances in machine learning and generative language models (e.g. ChatGPT) will solve many of these problems associated with the automatic retrieval of information from textual resources in the future. Meanwhile, simple text-mining approaches such as the one presented here offer a practical solution for retrieving information in a structured way.

## Supplementary Material

baae025_Supp

## Data Availability

The whole database is publicly available through the interactive web interface.
