# Supplementary material for: CoMentG: comprehensive retrieval of generic relationships between biomedical concepts from the scientific literature
Source: Database (Oxford). 2024 Apr 2;2024:baae025. doi: 10.1093/database/baae025 (PMC10986793; doi:10.1093/database/baae025)
Supplement: baae025_Supp [file baae025_supp.zip › suppl_data/Supp_file_1-Linkages_table.docx]

**CoMentG: Comprehensive retrieval of generic relationships between biomedical concepts from the scientific literature**

Jorge Novoa, Javier López-Ibáñez, Mónica Chagoyen, Juan A.G. Ranea, Florencio Pazos^*^

**Supplementary Material**

**Supplementary table 1.** For each of the 17 types of linkages, the number of relationships and number of terms involved are shown. The next two columns indicate whether there are resources with the same type of linkages, either using the same ontologies/vocabularies or using a different vocabulary, and also whether these relationships are obtained directly or indirectly (i.e. via an intermediate entity). The number of relationships in these other resources is also indicated when available.

| **Linkage** | **CoMentG**  **# relationships**  **(# terms)** | **Available using the same ontologies/vocabularies.**  **(direct/indirect)** | **Available using other ontologies/vocabularies.**  **(direct/indirect)** |
| --- | --- | --- | --- |
| GO - DOID | 247,398  (6,756 GOs)  (7,061 DOIDs) | No | Indirect relationships via chemical compounds, and using other disease vocabulary (GO-chem_comp-disease(MeSH)) (1).  753,000 links between 15,700 GO terms and 4,200 diseases. GO terms not restricted to human. |
| GO - MONDO | 531,210  (7,563 GOs)  (12,925 MONDOs) | No | Indirect relationships via chemical compounds, and using other disease vocabulary (GO-chem_comp-disease(MeSH)) (1).  753,000 links between 15,700 GO terms and 4,200 diseases. GO terms not restricted to human. |
| GO - HPO | 211,010  (7,187 HPOs)  (6,073 GOs) | Yes.  Indirect via shared genes (GO-gene-HPO) (2).  45,805 links (3,693 HPO, 2,801 GO) |  |
| GO - HMDB | 784,164  (7,645 GOs)  (20,506 HMDBs) | No | Direct. GO - chemical compound (MESHcomp) relationships used in (1) |
| GO - MESHcomp | 213,774  (2,893 MESHcomp)  (6,432 GOs) | Yes.  Direct. GO - chemical compound (MESHcomp) relationships used in (1). |  |
| MESHbac - DOID | 44,167  (4,259 MESHbac)  (2,447 DOIDs) | No | Many resources linking bacteria with disease. None using these vocabularies. |
| MESHcomp - DOID | 130,392  (2,880 MESHcomp)  (5,094 DOIDs) | No | Direct. chemical compound (MESHcomp) - diseases relationships used in (1). Diseases in other vocabulary. |
| MESHcomp - HPO | 139,630  (2,857 MESHcomp)  (5,222 HPOs) | No | Direct relationships between drugs (CID/Pubchem ids) and their side effects (UMLS ids) in SIDER (7).  139,756 linkages  1,430 drugs  5,868 side effects |
| HPO - HMDB | 328,892  (6,789 HPOs)  (15,670 HMDBs) | No | Direct relationships between drugs (CID/Pubchem ids) and their side effects (UMLS ids) in SIDER (7).  139,756 linkages  1,430 drugs  5,868 side effects |
| HMDB - DOID | 337,557  (17,123 HMDBs)  (6,733 DOIDs) | No | Many resources linking chemical compounds with diseases, using other disease nomenclature. E.g.  CTD (1) (Mesh_compound - Mesh_disease or OMIM);  KEGG (3) (KEGG compound - Disease);  HMDB (4) (HMDB - OMIM) |
| HMDB - MONDO | 828,504  (12,089 MONDOs)  (21,334 HMDBs) | No | Many resources linking chemical compounds with diseases, using other disease nomenclature. E.g.  CTD (1) (Mesh_compound - Mesh_disease or OMIM);  KEGG (3) (KEGG compound - Disease);  HMDB (4) (HMDB - OMIM) |
| HPO - CL | 43,062  (936 CLs)  (4,545 HPOs) | Yes.  Direct HPO-CL relationships in HPO’s .owl file (5). Few linkages.  221 HPO-CL  55 CL  219 HPO | No |
| DOID - CL | 51,430  (989 CLs)  (4,984 DOIDs) | No | No |
| HPO - UBERON | 707,203  (7,546 UBERONs)  (7,986 HPOs) | Yes.  Direct HPO-UBERON relationships in HPO’s .owl file (5).  5,707 linkages  1,136 UBERON  5,553 HPO | No |
| DOID - UBERON | 577,021  (7,450 UBERONs)  (7,697 DOIDs) | No | No |
| HPO - DOID | 594,110  (7,980 HPOs)  (7,896 DOIDs) | No | Direct.  HPO itself (5) includes the diseases associated to its clinical signs using diverse disease IDs (OMIM/Orphanet and  MONDO (6))  241,800 HPO-disease  10,446 HPOs  12,455 diseases |
| HPO - MONDO | 971,268  (14,455 MONDOs)  (8,062 HPOs) | Yes.  Direct. MONDO (6) includes linkages to HPO terms.  239,437 HPO-MONDO associations, including other HPOs apart from “phenotypic abnormality” |  |
|  |  |  |  |

1. Davis, A.P., Wiegers, T.C., King, B.L., et al. (2016) Generating Gene Ontology-Disease Inferences to Explore Mechanisms of Human Disease at the Comparative Toxicogenomics Database. PLOS ONE, 11, e0155530.
2. HPO2GO https://github.com/cansyl/HPO2GO
3. KEGG https://www.kegg.jp
4. HMDB https://hmdb.ca
5. HPO https://hpo.jax.org
6. MONDO <https://mondo.monarchinitiative.org/>
7. Kuhn M, Letunic I, Jensen LJ, Bork P. The SIDER database of drugs and side effects. Nucleic Acids Res. 2015
