# Supplementary material for: CoMentG: comprehensive retrieval of generic relationships between biomedical concepts from the scientific literature
Source: Database (Oxford). 2024 Apr 2;2024:baae025. doi: 10.1093/database/baae025 (PMC10986793; doi:10.1093/database/baae025)
Supplement: baae025_Supp [file baae025_supp.zip › SUPPLEMENTARY FILES.docx]

**SUPPLEMENTARY FILES**

**Supplementary File 1.** Table with detailed information on the 17 types of linkages. For each type of linkage, the number of relationships and number of terms involved are shown. The next two columns indicate whether there are resources with the same type of linkages, either using the same ontologies/vocabularies or using a different vocabulary, and also whether these relationships are obtained directly or indirectly (i.e. via an intermediate entity). The number of relationships in these other resources is also indicated when available.
